# Engagement with Nature and the Home Environment: Wellbeing and Proenvironmental Behavior among Irish and Italian University Students during the COVID-19 Emergency

**DOI:** 10.3390/ijerph20146432

**Published:** 2023-07-23

**Authors:** Iana Ivanova Tzankova, Catherine O’Sullivan, Alessandra Iva Facciuto, Luciana Sacchetti, Fabiana Fini, Elvira Cicognani, Annalisa Setti

**Affiliations:** 1 Department of Education Studies “G. M. Bertin”, University of Bologna, Via Filippo Re 6, 40126 Bologna, Italy; iana.tzankova2@unibo.it; 2 School of Applied Psychology, University College Cork, North Mall Enterprise Centre, T23 V2AY Cork, Irelanda.setti@ucc.ie (A.S.); 3 Planning & Communication Division, University of Bologna, Via Marsala 49, 40126 Bologna, Italy; 4 Workplace Health and Safety Service, University of Bologna, Largo Trombetti 3, 40126 Bologna, Italy; 5 Department of Psychology “R. Canestrari”, University of Bologna, Viale Berti Pichat 5, 40126 Bologna, Italy

**Keywords:** nature connectedness, time in nature, household chaos, wellbeing, proenvironmental behavior

## Abstract

Growing evidence shows that exposure to nature and psychological engagement with nature improve health and wellbeing and promote greater proenvironmental engagement. The unprecedented situation created by COVID-related lockdowns seems to have brought both potential distress with household confinements and greater research on experiences in nature. University students may have been particularly impacted as the quality of their home arrangements can vary substantially. The aim of the study was to examine how psychological engagement with nature (nature connectedness and noticing nature), time spent in nature, and household conditions relate to psychological wellbeing and proenvironmental behavior among university students. An online survey was administered to a sample of 566 university students from Italy and Ireland. Hierarchical multiple regressions were performed to investigate the relationships between variables. The results indicate that time spent in nature and psychological engagement with nature in terms of nature connectedness and noticing nature were associated with increased wellbeing and pro-nature-conservation behavior, controlling for demographic covariates. Moreover, the perception of chaos in one’s household was related to decreased wellbeing during the prolonged COVID-19 emergency. The findings highlight the need to invest in accessible natural places for students and to focus campus sustainability practices on encouraging nature connectedness to promote wellbeing and proenvironmental engagement.

## 1. Introduction

### 1.1. Nature Connectedness, Time in Nature, and the Home Environment: Associations with Wellbeing and Proenvironmental Behavior among University Students in Ireland and Italy

In response to the global emergency related to the transmission of the coronavirus SARS-CoV-2, authorities in many countries established public health control measures, including national lockdowns and limitations to movement during one or more periods during the COVID-19 pandemic. These measures confined individuals in their homes for extended periods of time, impacting their experience of household and outdoor spaces with important consequences for health and wellbeing [1]. University students’ lives were deeply impacted with an abrupt transition to online learning, social distancing from friends and home confinement bringing negative emotional experiences, in addition to COVID-related stress and growing anxiety and depression [2,3]. It is of great interest to better understand how students’ relationships with nature served as a buffer for wellbeing, while their living conditions at home may have been unfavorable due to the lockdown. Adding to previous studies [4], the present research examines how household conditions during the pandemic were associated with wellbeing in conjunction with nature connectedness, visits, and time spent in nature. We also examine whether the novel situation created by the lockdown, with a potential increase in noticing nature and experiencing nature contact, may have improved proenvironmental behavior.

### 1.2. Benefits of Exposure and Connection to Nature

There is growing evidence that exposure to nature improves health and wellbeing [5,6]. Studies published in the last two years have also evidenced beneficial effects of nature exposure and outdoor activities during the pandemic for young people [6,7]. During the lockdowns and restrictions related to COVID-19, experience of nature has been varied; it generally increased, and most evidence suggests it improved mental health and life satisfaction [8]. As everyday life was disrupted, people seem to have engaged with nature more and increased their awareness of nature-related topics and places [9].

Both the time spent in nature (exposure) and the psychological connection with nature are key aspects of the human–nature relationship [4]. Recent studies have shown that visits to and spending time in nature are associated with greater mental wellbeing [10,11]. Psychological engagement with nature has also been found to associate with greater wellbeing through noticing nature and feeling a connection with it [4,12,13]. Indeed, Richardson and colleagues have argued that it is important to assess psychological engagement and relationship with nature as essential for wellbeing [4], rather than solely visits and time in nature in a “dose–response” perspective [14].

Another benefit of an increased human–nature relationship is that it can promote and motivate greater proenvironmental engagement in individuals. Nature connectedness and noticing nature in the moment have been identified as important predictors of pro-nature-conservation behaviors beyond visits to nature [13,15].

### 1.3. Household Conditions during the COVID-19 Pandemic

Since lockdowns and social distancing measures confined more people in their homes, individuals’ household living conditions may have had an important role for wellbeing and prosocial choices [16]. Recent research suggests that home confinement during the COVID-19 pandemic has had negative influence on individual health and wellbeing [17]. A potential risk factor for health and wellbeing within the home environment has been identified in developmental psychology as *household chaos*, or daily disruptions within the household [18]. It includes unpredictability, crowding, and noise in the household [19]. Consistent household chaos can compromise wellbeing and healthy behaviors [20]. Changes during the pandemic may have contributed to an added household chaos with negative impact on adults as well [21]. Existing studies have overlooked the potential role of the quality of the household environment in young adults’ wellbeing and proenvironmental behavior. We seek to address such gaps by exploring how perceived disruptions in household conditions along with nature exposure and engagement are associated with these outcomes. To our knowledge, this is the first to study to investigate the roles of engagement with nature and perceived household conditions simultaneously, and whether the potential benefits of the former can offset the potential drawbacks of the latter.

### 1.4. The Present Research

The purpose of the study is to examine the relationship of psychological engagement with nature (nature connectedness and noticing nature), time spent in nature, and the household environment with psychological wellbeing and pro-nature-conservation behavior. Based on the existing literature, we expect that nature connectedness, current engagement with nature, and time spent in nature are associated positively with both wellbeing and conservation behavior. Moreover, we hypothesize perceived household chaos to be negatively associated with wellbeing. Since there is a lack of theoretical development and evidence of the potential relation between household conditions and youth environmental behavior, no specific hypothesis was advanced, but the association was explored as an open research question. 

## 2. Methods

### 2.1. Participants and Procedure

The research was approved by the ethics committees of the University of Bologna and the University College Cork. Students from the two universities were invited to participate in the study through institutional e-mails and social media posts on the universities’ pages. The data were collected in June–July 2021, at the tail-end of an academic year marked by the COVID-19 emergency and intermittent lockdowns and restrictions. Participants were provided with consent forms before filling the survey online. The instrument was anonymous. Surveys with missing information and incomplete responses were excluded (28.9% of the original sample).

The sample consisted of 566 participants from Italy (*N* = 456, 80.6%) and Ireland (*N* = 110, 19.4%). Female respondents were overrepresented (Italy: 78.5% female, 21.5% male; Ireland: 71.8%, 28.2%), which may be partially related to the prevalence of female students in the universities. Respondents who indicated their gender as nonbinary or third gender were excluded from the analyses, due to the low number impeding comparisons (*N* = 8). Respondents’ age ranged between 18 and 65 years old, with 82.1% of the respondents between the ages of 18 and 29 years old, and 17.9% between 30 and 65 years old (6.2% were between 42 and 65 years old). Participants reported living in households where money largely covered their family needs (*M* = 3.92, *SD* = 1.13, Min = 1, Max = 5). 

### 2.2. Measures

The survey was developed as part of the research project “Green Spaces & Wellbeing” within the collaboration agreement between the University of Bologna (Italy) and the University College Cork (Ireland) in the context of the Greenmetric World University Ranking Network. 

#### 2.2.1. Independent Variables

Demographic information. Participants were asked to report their age, gender, and perceived family economic situation (“Does the money your household cover everything your family needs?”; 1 = *not at all* to 5 = *fully*) [22].

Nature connectedness. Nature connectedness was assessed with the item “I feel part of nature” from the Nature Connection Index (NCI) [23], which was measured on a 7-point Likert scale (1 = *completely disagree* to 7 = *completely agree*). 

Engagement with nature. Current nature engagement was assessed with the item “I am taking more time to notice and engage with everyday nature (e.g., listening to birdsong, noticing butterflies)” [4] measured on a 7-point Likert scale (1 = *completely disagree* to 7 = *completely agree*). 

Time spent in nature. Participants were asked “How many times have you been outdoors in green areas and natural places in the last 14 days? (Trips include “green areas in the city/country”, “in the countryside”, “on the coast”, but do not include “time spent in your own garden”, “time spent outside on business” or “time spent abroad”)” [4]. They had to enter the number of times outdoors.

Household conditions. The perceived disruptions in household conditions were measured with 14 items from the Confusion, Hubbub and Order Scale (CHAOS) [19], assessing daily confusion and disruption to organization within the home setting. An example question was “No matter what our family plans, it usually does not seem to work out” [19]. Response options were 1 = *true* or 0 = *false*, with reverse coding for six items. Scores were summed and higher scores indicated a more chaotic environment. The reliability of the scale was good (α = 0.73).

#### 2.2.2. Dependent Variables

Wellbeing. Wellbeing was assessed with 4 items measured on a 10-point Likert scale (0 = *not at all* to 10 = *fully*), which asked participants about their satisfaction with life, sense of worthwhile life, and their feelings of happiness and loneliness (reversed score). These were items used in previous studies on the topic to assess constructs associated with wellbeing [4,24,25]. An example item was “Overall, how satisfied are you with your life nowadays?” [4,24,25]. The reliability was good (α = 0.79).

Pro-nature-conservation behavior (PNCB). The 8-item short-form Pro-Nature Conservation Behavior Scale (ProCoBS) [26] was used to assess engagement in activities preserving and enhancing biodiversity. Items were measured on a 7-point Likert scale (1 = *never* to 5 = *always*). An example was “I sign petitions supporting nature conservation efforts” [26]. The reliability was good (α = 0.76).

## 3. Results

### 3.1. Descriptive Statistics and Correlations between Variables

The means and standard deviations of the main variables in the analyses are reported in Table 1. The bivariate correlations between all variables are shown in Table 2.

As shown in Table 2, there were significant correlations between the main independent variables (nature connectedness, engagement with nature, time in nature, and perceived household conditions) and the dependent variables, apart from household conditions and pronature behavior. 

Differences were found between the Italian and the Irish sample in age (F_(1,563)_ = 21.107, *p* < 0.001), time spent in nature (F_(1,556)_ = 5.756, *p* = 0.017) and perceived household conditions (F_(1,563)_ = 57.423, *p* < 0.001). 

As shown in Table 2, the Italian participants were younger, spent less time in nature, and perceived their households as less chaotic than the Irish students. To better understand whether the difference in perceived household conditions could be related to different housing in Italy and in Ireland, we explored students’ housing characteristics. However, the two samples did not differ significantly in the number of people within the household and in the perceived satisfaction with their house. There were some differences regarding the type of housing and the presence of a private garden or courtyard—the majority in Ireland lived in a house (85.5%) and a very high percentage had a private garden (90%), while the majority in Italy lived in multi-room flats (60.7%) and slightly fewer students had gardens or courtyards (71%). 

### 3.2. Regression Analyses

A series of hierarchical multiple regressions were performed to investigate the relationships between nature connectedness, time and engagement with nature, perceived household conditions, wellbeing, and pro-nature-conservation behavior. 

For each dependent variable, there were three blocks of independent variables: the initial block consisted of demographic variables (country, age, gender, and family economic situation); nature connectedness, time spent in nature and engagement with nature were inserted in the second block; and finally, household conditions constituted the third block. The overall sample size provided adequate power for eight independent variables: at α = 0.05 and power = 0.80, a sample size of 109 was sufficient for a medium effect size. The assumptions for multicollinearity, independence of errors and homoskedasticity were satisfied (VIF = 1.005–1.170; tolerance = 0.854–0.995; Durbin–Watson = 1.918–2.110). 

Table 3 reports the regression coefficients for the effects in the complete model on both dependent variables in the overall sample.

#### 3.2.1. Wellbeing

It was found that family economic situation, nature connectedness, current engagement with nature, and household conditions were significantly related to wellbeing (F_(8,546)_ = 10.593, *p* < 0.001, R^2^ = 0.134, R^2^_Adj._ = 0.122). Participants whose families were wealthier had higher wellbeing. Feeling as part of nature and taking time to notice nature were also related to higher wellbeing. Perceived disruptions in household conditions were negatively related to wellbeing.

#### 3.2.2. Pro-Nature-Conservation Behavior

Age, gender, nature connectedness, current engagement with nature, and time spent in nature were significantly related to pro-nature-conservation behavior (F_(8,546)_ = 11.240, *p* < 0.001, R^2^ = 0.141, R^2^_Adj._ = 0.129). In particular, older participants were more likely to engage in pronature behavior, while male participants were less likely. Feeling as part of nature, taking time to notice nature and spending time in natural spaces were all strongly and positively related to PNCB. Household conditions did not contribute to explaining PNCB.

#### 3.2.3. Comparison between Italy and Ireland

The same hierarchical multiple regression analyses were also performed for each country separately. The sample sizes provided adequate power for seven independent variables: at α = 0.05 and power = 0.80, a sample size of 102 was sufficient for a medium effect size. The assumptions for multicollinearity, independence of errors, and homoskedasticity were satisfied (VIF = 1.001–1.487; tolerance = 0.672–0.999; Durbin–Watson = 1.842–2.206). Table 4 reports the regression coefficients for the effects on both dependent variables within the Italian and Irish samples.

The results suggest that there were differences between the Italian and the Irish sample in the relevance of some of the independent variables for both wellbeing and PNCB. Within the Italian sample, family economic situation, nature connectedness, and household conditions were significantly related to wellbeing (F_(7,441)_ = 8.364, *p* < 0.001, R^2^ = 0.117, R^2^_Adj._ = 0.103). Within the Irish sample, wellbeing was significantly related to engagement with nature, time spent in nature, and household conditions (F_(7,98)_ = 8.351, *p* < 0.001, R^2^ = 0.374, R^2^_Adj._ = 0.329).

Among Italian participants, PNCB was associated with age, gender, family economic situation, nature connectedness, and engagement with nature (F_(7,441)_ = 10.367, *p* < 0.001, R^2^ = 0.141, R^2^_Adj._ = 0.128). Among Irish participants, PNCB was significantly related only to time spent in nature (F_(7,98)_ = 5.300, *p* < 0.001, R^2^ = 0.275, R^2^_Adj._ = 0.223).

## 4. Discussion

The present research extended previous work by examining the role of nature exposure, connection and engagement with nature, and perceived disruption in household conditions simultaneously as factors in wellbeing and pro-nature-conservation outcomes. The results indicate that time spent in nature and psychological engagement with nature in terms of sense of connectedness and noticing nature were associated with increased wellbeing and pro-nature-conservation behavior, controlling for demographic covariates. Moreover, the perception of chaos in one’s household was also related to wellbeing during the prolonged COVID-19 emergency. 

The findings that increased nature connectedness and current engagement and time in nature were related to wellbeing and pronature behavior are largely consistent with previous research in the field [4,13,24]. Past studies have been inconsistent as to the relative importance of psychological engagement with nature in comparison to direct exposure to nature (spending time in natural spaces). For example, Martin et al. [13] found that nature connectedness was related to wellbeing outcomes and conservation behavior, but visits to nature were not. The authors suggested that there were moderating effects, in which visits were associated with wellbeing only for individuals who felt less connected to nature while the opposite effect was observed for conservation behavior. Using a wider span of time for the measure of exposure (a year vs. a week); however, Richardson and Hamlin [4] found both factors to be consistent predictors of wellbeing and conservation behavior outcomes. Our results also suggest some inconsistencies regarding the specific aspects of the relationship with nature associated with these outcomes. While we found overall significant relationships of nature connectedness and current engagement with wellbeing, we also found differences among the Italian and Irish subsamples. Nature connectedness was associated with higher wellbeing among Italian students, while current engagement and time in nature were significant predictors of greater wellbeing among Irish students. With respect to conservation behavior, however, psychological connection and engagement were important among Italian students and time spent in nature—among Irish students. These findings suggest that psychological connection and nature exposure have different importance in the different national contexts. It is possible that characteristics related to the availability, types, and quality of green and natural spaces in the two settings moderate the effects by enhancing psychological connection for Italian students and time spent in nature for Irish students. The complexity of these results suggests that contextual factors (e.g., presence, accessibility, characteristics and quality of natural places, social norms, cultural factors, etc.) may have a role in determining the way of engaging with nature that is more relevant for higher wellbeing and proenvironmental behavior. Indeed, recent theoretical developments adopt a complex multidimensional dynamic approach of the interaction between nature and health, which considers subjective aspects of both individuals (cultural, social and personal modifiers) and the natural environments (perceived characteristics and quality) [27]. It follows that further research should provide a more extensive examination of possible contextual moderators of the relationships between green spaces, environmental behavior, and wellbeing. 

A novel contribution of this study was the examination of perceived household disruptions among young people during the pandemic and its role in wellbeing and environmental engagement alongside contact with nature. As public health measures restricted movement and determined a shift to home-based online learning, the household environment took center stage for university students. Our results suggest that a negative perception of the levels of noise, confusion and crowdedness in the household are associated with an important decrease in wellbeing. This was confirmed for both Italian and Irish students. While this finding is novel, it is broadly in line with research within developmental psychology on the impacts of household chaos to families and child health [20,21]. The results highlight the importance of considering students’ home environments, especially during stressful events such as the pandemic, in assessing their wellbeing needs. Other research with Italian students from Bologna has also shown that during the COVID-19 crisis, difficulties of finding an adequate space to study without interruptions was an important factor for students’ wellbeing [28]. It is interesting to consider whether connection with nature and the use of green spaces can be a source of refuge and coping with the effect of disruption and confusion in the home environment. In our results among Irish students, the contribution of nature engagement and visits was bigger than that of household conditions, suggesting that in some contexts, exposure to nature and behavior change through noticing nature can offset negative home environments. Given the novelty of these findings, more research is needed to determine what may underpin these results and in what ways students can cope with household chaos positively. More research is also needed to better understand cross-country differences in the psychological experiences related to one’s household, as our results suggest that Irish students experienced their home organization more negatively even though housing conditions (e.g., number of people) and satisfaction did not differ between the two countries. 

Finally, our findings point to some sociodemographic differences in the level of proenvironmental behavior, particularly among Italian students. In line with previous research [29,30], we found women to be more likely to engage in proconservation behavior. This effect has been related to gender role socialization and the tendency of women to engage in civic and volunteering activities in line with nurturing and caregiving gender expectations [31,32]. We also found that older participants were involved more in such behavior. Previously, it has been suggested that such a positive age effect can be partially explained by knowledge accumulated over time [33]. However, overall, research has been inconclusive on the direction of age and generational differences in environmental concern and behavior, as some studies suggest younger people are more environmentally friendly, while other studies have found the inverse or no age differences in behavior [34]. It has been suggested that biospheric values and political orientation might be more robust predictors in this sense, while other factors that function as barriers might hinder the actual engagement in behavior [34,35]. It is thus possible that the age differences we found could be explained by other internal and/or contextual factors. 

Several limitations of this study should be mentioned. First, the analyses were based on cross-sectional survey data, which limited any causal inference for the identified associations. Further research should employ longitudinal designs that can show the direction of the studied effects, as well as shed light on possible long-term impacts of nature engagement and household conditions. Second, the results were based on nonrepresentative samples of university students and cannot be generalized to the student or general population. Third, the data were based on self-reports, which can be subject to recall bias. Fourth, our measures did not consider the specific characteristics and quality of natural places that participants were exposed to and future studies could address these aspects. A potential limitation to the measure of nature exposure is that it excluded the time spent in one’s private garden. Recent studies during COVID-19 suggest that private gardens could have important contributions to feelings of nature connectedness and wellbeing [36]. Moreover, future research on the topic can explore mediating and moderating factors with more robust samples, for example clarifying the possible interactions between life circumstances, household conditions and experience of nature. As our findings suggest, future studies should also further explore the psychological experience of the home environment among university students and possible cross-country and contextual differences that can shed light on the factors that benefit or hinder students’ wellbeing in relation to their household conditions.

## 5. Conclusions

The study has significant implications for environmental policies in universities. The findings highlight the need to invest in accessible natural places for students to promote wellbeing and environmental engagement. Beyond providing spaces, however, the results on the importance of psychological engagement with nature suggest that interventions and education should focus on encouraging nature connectedness and increasing the noticing of nature, which will be an important component of preparedness for the future. Moreover, such interventions should also consider assessing students’ household conditions and their impact on students’ wellbeing to better respond to their need for green and restorative spaces.

## Figures and Tables

**Table 1:** Means and standard deviations of the main variables.

	Italy		Ireland		Overall	
	*M*	*SD*	*M*	*SD*	*M*	*SD*
Nature connectedness	5.910	1.380	5.800	1.012	5.890	1.317
Engagement with nature	5.200	1.683	5.420	1.480	5.240	1.647
Time in nature	6.590	5.023	7.981	6.743	6.857	5.417
Household conditions	4.781	2.746	6.827	1.400	5.178	2.666
Wellbeing	5.457	1.550	5.505	1.773	5.496	1.731
PNCB	2.898	0.928	2.769	0.981	2.873	0.939

Note. PNCB = pro-nature-conservation behavior.

**Table 2:** Correlations between the variables.

	1.	2.	3.	4.	5.	6.	7.	8.
1. Country								
2. Age	−0.179 ***							
3. Gender	−0.067	0.024						
4. Family economic situation	−0.006	−0.068	−0.011					
5. Nature connectedness	0.036	0.074 *	−0.035	0.004				
6. Engagement with nature	−0.045	0.027	−0.080 *	0.008	0.363 ***			
7. Time in nature	−0.101 **	0.024	0.006	0.020	0.072 *	0.048		
8. Household conditions	−0.299 ***	−0.035	−0.010	−0.146 ***	−0.029	0.005	0.048	
9. Wellbeing	0.051	0.073 *	−0.036	0.135 **	0.191 ***	0.173 ***	0.085 *	−0.261 ***
10. PNCB	0.066	0.094 *	−0.156 ***	−0.060	0.273 ***	0.238 ***	0.121 **	−0.031

Note. * *p* < 0.05, ** *p* < 0.01, *** *p* < 0.01. Country: 0 = Ireland, 1 = Italy; Gender: 0 = Female, 1 = Male; PNCB = pro-nature-conservation behavior.

**Table 3:** Multiple regression analysis on wellbeing and PNCB: overall sample.

	Wellbeing		PNCB	
	*β*	*p*	*β*	*p*
Country (Italy)	−0.005	0.901	0.078	0.071
Age	0.056	0.174	0.085	0.037
Gender (male)	−0.025	0.534	−0.135	0.001
Family economic situation	0.099	0.014	−0.061	0.128
Nature connectedness	0.129	0.003	0.196	0.000
Engagement with nature	0.119	0.006	0.153	0.000
Time in nature	0.078	0.052	0.108	0.007
Household conditions	−0.247	0.000	−0.016	0.713

Note. *β* are standardized. Country: 0 = Ireland, 1 = Italy; gender: 0 = female, 1 = male; PNCB = pro-nature-conservation behavior.

**Table 4:** Multiple regression analysis on wellbeing and PNCB: Italy and Ireland.

	Wellbeing		PNCB	
	*β* Italy	*β* Ireland	*β* Italy	*β* Ireland
Age	0.081	−0.060	0.145 **	−0.043
Gender (male)	−0.037	0.024	−0.156 ***	−0.110
Family economic situation	0.097 *	0.079	−0.106 *	0.089
Nature connectedness	0.134 **	0.046	0.201 ***	0.121
Engagement with nature	0.083	0.404 ***	0.142 **	0.188
Time in nature	0.037	0.222 *	0.028	0.329 **
Household conditions	−0.232 ***	−0.208 *	−0.013	0.022

Note. * *p* < 0.05, ** *p* < 0.01, *** *p* < 0.01. *β* are standardized. Gender: 0 = female, 1 = male; PNCB = pro-nature-conservation behavior.

## Data Availability

The data presented in this study are openly available in OSF at https://doi.org/10.17605/OSF.IO/9G2MP (accessed on 21 July 2023), reference number 9G2MP [37].
